# *In vitro* comparative study of provisional crown and bridge materials fabricated via 3D printing, milling and traditional techniques

**DOI:** 10.6026/973206300220985

**Published:** 2026-02-28

**Authors:** Preeti Mankar, Manoj Sakhare, Rajasekar M, Shalvika Khavnekar, Swati Pandey, Sukhjit Kaur

**Affiliations:** 1 Department of Prosthodontics, Crown and Bridge Implantology, Nanded Rural Dental College, Research Centre, Nanded, Maharashtra, India; 2 Department of Oral and Maxillofacial Surgery, ESIC Dental College and Hospital, Kalaburagi, India; 3 Department of Prosthodontics and Crown & Bridge, Punjab Government Dental College and Hospital, Amritsar, Punjab, India

**Keywords:** CAD/CAM milling, marginal adaptation, provisional restorations, three-dimensional printing, traditional fabrication

## Abstract

Despite widespread clinical use of provisional crown and bridge restorations, there is a significant gap in comparative evidence regarding the influence of different fabrication techniques (3D printing, CAD/CAM milling and conventional methods) on their mechanical and marginal performance. This *in vitro* study systematically compares key properties of provisional restorations fabricated using these three techniques to generate objective, standardized data for clinical evaluation. The findings provide evidence-based insight into the relative advantages of digital fabrication methods, particularly CAD/CAM milling, in terms of predictability and durability. This study advances the knowledge by offering clinicians clearer guidance on selecting the most reliable approach for provisional restorations.

## Background:

Provisional crown and bridge restorations play a pivotal role in fixed prosthodontic treatment by protecting prepared teeth, maintaining occlusal relationships, preserving periodontal health and ensuring patient comfort and aesthetics during the interim phase before definitive prosthesis delivery [1]. An ideal provisional restoration should demonstrate adequate mechanical strength, marginal accuracy, color stability, surface smoothness and biocompatibility, while also being easy to fabricate and repair [2]. Traditionally, provisional crowns and bridges have been fabricated using direct or indirect techniques with materials such as polymethyl methacrylate (PMMA), polyethyl methacrylate (PEMA) and bis-acryl composite resins. Although widely used, these conventional materials and techniques are often associated with limitations such as polymerization shrinkage, heat generation, marginal discrepancies, porosity and variability in mechanical properties due to operator- dependent factors [3]. With the rapid advancement of digital dentistry, computer-aided design and computer-aided manufacturing (CAD/CAM) technologies have transformed the fabrication of provisional restorations. Subtractive manufacturing through milling of pre-polymerized PMMA blocks offers improved mechanical strength, enhanced marginal adaptation and reduced polymerization defects compared to chairside-fabricated conventional provisionals [4]. However, milling techniques are associated with material wastage, higher equipment costs and limitations in reproducing complex internal geometries. Additionally, the subtractive process may induce internal stresses and microcracks depending on milling parameters and bur wear [5]. Additive manufacturing, commonly known as three-dimensional (3D) printing, has emerged as a promising alternative for the fabrication of provisional crowns and bridges. 3D printing technologies such as stereolithography (SLA), digital light processing (DLP) and material jetting enable layer-by-layer fabrication of restorations with high precision, reduced material wastage and efficient reproduction of complex anatomical details [6].

Printed provisional materials are typically light-cured resin-based polymers specifically formulated for interim restorations. These materials have shown potential advantages in terms of dimensional accuracy, surface finish and consistency due to controlled manufacturing processes. Nevertheless, concerns remain regarding their mechanical properties, degree of conversion, wear resistance and long-term clinical durability, particularly when compared with milled and conventionally fabricated provisional materials [7]. Despite the increasing clinical adoption of digital workflows, there is ongoing debate regarding the comparative performance of provisional restorations fabricated using 3D printing, milling and traditional techniques [8]. Variations in fabrication method, material composition, polymerization mode and post-processing protocols may significantly influence properties such as flexural strength, fracture resistance, marginal fit, surface roughness and color stability. *In vitro* studies provide a controlled environment to systematically evaluate and compare these parameters without the confounding factors present in clinical settings. Such comparative assessments are essential to guide clinicians in evidence-based material selection and technique choice for provisional restorations [9]. Therefore, it is of interest to evaluate provisional crown and bridge materials fabricated via 3D printing, milling and traditional techniques is clinically relevant and timely.

## Methodology:

This *in vitro* comparative study aimed to evaluate the mechanical and marginal performance of provisional crown and bridge restorations fabricated using three different techniques: 3D printing, CAD/CAM milling and conventional methods. A total of 210 samples were used, with 70 samples from each fabrication group. Each group was further divided into five sub-groups of 14 samples each, based on the properties to be evaluated: marginal adaptation (MA), fracture resistance (FR), flexural strength (FS), surface roughness (SR) and color stability (CS). For the fabrication of the provisional restorations, Group I samples were created using 3D printing with Next Dent C&B Micro Filled Resin (Vertex-Dental, Netherlands), featuring a layer thickness of 50 µm. Group II samples were fabricated using CAD/CAM milling from pre-polymerized PMMA blocks (Ivoclar PMMA CAD/CAM Disc, Liechtenstein). Group III samples were created using conventional fabrication methods with Protemp™ 4 Temporization Material (3M ESPE, USA). Following fabrication, all samples were subjected to standardized testing protocols. Marginal adaptation (MA) was measured using a stereo-microscope (Leica M80, Germany) at x40 magnification, with measurements taken at four points on each sample: mid-buccal, mid-lingual, mid-mesial and mid-distal. Fracture resistance (FR) was tested using a Universal Testing Machine (Instron 3366, USA), with a vertical load applied at 1 mm/min until fracture occurred. Flexural strength (FS) was evaluated using a three-point bending test on the Universal Testing Machine, following ISO 4049 specifications. Surface roughness (SR) was measured using a contact-type profilometer (Mitutoyo SJ-210, Japan), calculating the mean Ra value from three passes. Finally, color stability (CS) was assessed using a reflectance spectrophotometer (VITA Easyshade V, Germany) before and after artificial aging, with color changes (ΔE) calculated using the CIE Lab* formula. The data collected from these tests was analyzed for statistical significance using ANOVA, with a p-value of <0.05 considered statistically significant for all parameters. This comprehensive approach ensured that the properties of each fabrication technique were rigorously evaluated and compared.

## Results:

The results of the present *in vitro* comparative study demonstrated statistically significant differences in the mechanical and physical properties of provisional crown and bridge materials fabricated using 3D printing, CAD/CAM milling and conventional techniques. All tested parameters were analyzed across the three groups and the findings were summarized. Marginal adaptation analysis revealed that CAD/CAM milled provisional restorations exhibited the lowest mean marginal gap values, followed by 3D printed restorations, while conventionally fabricated provisionals showed the highest marginal discrepancies. The difference among the three groups was statistically significant (p < 0.05), indicating superior marginal accuracy with digitally fabricated techniques (Table 1). Fracture resistance testing showed that milled PMMA provisional restorations demonstrated the highest mean fracture resistance, with values significantly greater than both 3D printed and conventional groups (p < 0.001). The 3D printed group exhibited intermediate fracture resistance, whereas conventionally fabricated provisionals recorded the lowest resistance to fracture (Table 2). Flexural strength evaluation followed a similar trend. CAD/CAM milled provisionals showed the highest flexural strength, followed by 3D printed provisionals, while conventional bis- acryl provisionals exhibited significantly lower flexural strength values. These differences were statistically significant (p < 0.001), as presented in (Table 3). Surface roughness assessment revealed that 3D printed provisional restorations had comparatively smoother surfaces than conventionally fabricated provisionals, though milled restorations demonstrated the lowest surface roughness values overall. The difference between all three groups was statistically significant (p < 0.05) (Table 4). Color stability evaluation after artificial aging showed that milled provisional restorations had the least color change (ΔE), followed by 3D printed restorations. Conventional provisional materials demonstrated the highest color alteration, indicating inferior color stability. The intergroup differences were statistically significant (p < 0.05) (Table 5). Overall, digitally fabricated provisional restorations, particularly CAD/CAM milled provisionals, exhibited superior performance across most evaluated parameters when compared with 3D printed and conventional techniques.

## Discussion:

The present in vitro study compared provisional crown and bridge materials fabricated using 3D printing, CAD/CAM milling and conventional techniques and demonstrated that digitally fabricated provisional restorations exhibited superior mechanical and physical properties when compared with conventionally fabricated provisionals. Among the digital techniques, CAD/CAM milled provisional restorations consistently showed the most favorable outcomes across all evaluated parameters, followed by 3D printed restorations. Marginal adaptation is a critical determinant of the clinical success of provisional restorations, as marginal discrepancies may lead to plaque accumulation, gingival inflammation and microleakage. In the present study, CAD/CAM milled provisionals showed the least marginal gap, which can be attributed to the use of pre-polymerized PMMA blocks and precise subtractive manufacturing processes [10]. This finding aligns with Pallis *et al.* (2025) [11], who reported that CAD/CAM- fabricated materials demonstrated superior surface and mechanical properties relative to 3D-printed and conventional counterparts in a comparative *in vitro* analysis of provisional materials. Several studies have explored similar parameters. For instance, Digholkar *et al.* (2016) [12] investigated flexural strength and microhardness of provisional materials manufactured by rapid prototyping, CAD/CAM and conventional techniques, finding significant differences among fabrication methods, with milled specimens often showing more favorable mechanical properties than 3D printed or conventional groups. This supports our observation that digitally fabricated provisionals can offer enhanced property consistency. In contrast, the study by Botadra *et al.* (2024) [13] on interim fixed dental prostheses fabricated via conventional, 3D printing and CAD/CAM milling methods found variations in both marginal gap and flexural strength depending on the fabrication technique, indicating that each modality has specific strengths and limitations that can influence clinical decision-making. While our results showed milled restorations to have the lowest marginal discrepancies, Botadra *et al.* highlighted that design protocols can influence marginal outcomes even for 3D printed materials.

The superior marginal adaptation and mechanical consistency of milled provisionals in our study may also reflect improvements in digital scanning and CAD workflows, which reduce operator variability compared to manual fabrication. These digital advantages parallel findings in restorative dentistry literature demonstrating that CAD/CAM processes often yield restorations with tighter tolerances and improved mechanical profiles when compared with conventional methods [14]. However, 3D printed materials should not be discounted: their ability to reproduce complex geometries rapidly and with minimal material waste makes them highly relevant in contemporary digital workflows [15]. Improvements in resin formulations and post-processing protocols may further enhance their performance, narrowing the gap with milled provisionals in future iterations [16].

## Conclusion:

We show that provisional crown and bridge restorations fabricated using digital techniques exhibited superior performance compared with conventional methods. CAD/CAM milled provisional restorations showed the best marginal adaptation, mechanical strength, surface smoothness and color stability, followed by 3D printed restorations. Thus, we show the preferential use of CAD/CAM milling for achieving predictable and durable provisional restorations in contemporary prosthodontic practice.

## Figures and Tables

**Table 1:** Comparison of marginal adaptation among study groups

**Group**	**Mean Marginal Gap (µm)**	**SD**	**p-value**
3D Printed	92.4	8.6	
CAD/CAM Milled	68.7	7.9	<0.05
Conventional	124.3	10.2	

**Table 2:** Comparison of fracture resistance among study groups

**Group**	**Mean Fracture Resistance (N)**	**SD**	**p-value**
3D Printed	842.5	75.4	
CAD/CAM Milled	1126.8	88.6	<0.001
Conventional	615.9	69.2	

**Table 3:** Comparison of flexural strength among study groups

**Group**	**Mean Flexural Strength (MPa)**	**SD**	**p-value**
3D Printed	96.3	6.8	
CAD/CAM Milled	128.4	7.5	<0.001
Conventional	74.6	5.9	

**Table 4:** Comparison of surface roughness values among study groups

**Group**	**Mean Surface Roughness (Ra,µm)**	**SD**	**p-value**
3D Printed	0.48	0.05	
CAD/CAM Milled	0.36	0.04	<0.05
Conventional	0.62	0.06	

**Table 5:** Comparison of color stability (ΔE) among study groups

Group	Mean ΔE Value	SD	p-value
3D Printed	2.14	0.32	
CAD/CAM Milled	1.58	0.27	<0.05
Conventional	3.46	0.41	
